# Formation and physical properties of the self-assembled BFO–CFO vertically aligned nanocomposite on a CFO-buffered two-dimensional flexible mica substrate

**DOI:** 10.1039/d1ra01158h

**Published:** 2021-04-27

**Authors:** Tahta Amrillah, Angga Hermawan, Shu Yin, Jenh-Yih Juang

**Affiliations:** Department of Nanotechnology, Faculty of Advanced Technology and Multidiscipline, Universitas Airlangga Surabaya 60115 Indonesia tahta.amrillah@ftmm.unair.ac.id; Department of Electrophysics, National Chiao Tung University Hsinchu 30010 Taiwan jyjuang@cc.nctu.edu.tw; Institute of Multidisciplinary Research for Advanced Material (IMRAM), Tohoku University 2-1-1 Katahira, Aoba-Ku Sendai Miyagi 980-8577 Japan

## Abstract

Engineering the interfaces between materials of different structures and bonding nature in a well-controlled fashion has been playing a key role in developing new devices with unprecedented functionalities. In particular, direct growth of nanostructures on van der Waals substrates not only is essential for fully exploiting the potential of a wide variety of self-assembled nano-sized heterostructures but also can expand the horizons for electronic and photonic applications that involve nanostructures of specific composition and geometry. In the present work, we demonstrate the epitaxial growth of a self-assembled vertically aligned nanocomposite of magnetoelectric oxides on a flexible substrate *via* van der Waals epitaxy, which evidently adds an additional dimension of flexibility to similar thin-film heteroepitaxy architectures that have been mostly realized on rigid lattice-matched substrates. It is noted that the utilization of buffer layers is essential for obtaining high-quality flexible thin films with vertically aligned nanocomposite architecture. We believe that this route can provide alternative options for developing flexible thin-film devices with heteroepitaxy architectures of other functional materials.

## Abstract 2

BiFeO_3_–CoFe_2_O_4_ vertically aligned nanocomposites, which mainly discovered in thin-films deposited on rigid substrates, have been successfully transformed into a flexible thin-film using a mica substrate.

## Graphical abstract

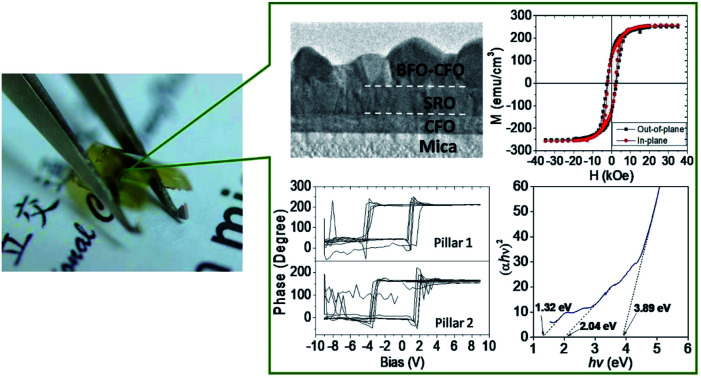

## Introduction

With the exponentially increasing demands in speed and functionalities for meeting the rapidly moving technologies, integrating materials with vastly different structures and properties onto the same substrate, preferably flexible in many cases, to make the desired devices not only has become mainstream research but a necessity.^1–4^ Indeed, ubiquitous applications of flexible electronics have been forecasted or even realized in various industry sectors, such as wearable healthcare and environmental monitoring, human-machine interactivity, displays and storage devices, energy conversion and harvesting, communication, and wireless networks.^5–8^ Numerous functional materials which were originally delivered in thin films deposited on rigid substrates have been transformed into flexible thin films to meet the demands of flexibility while keeping the functionality required for device applications.^5–8^ In this respect, the selection of proper flexible substrate is of pivot importance. Nevertheless, such choices are often very limited due to strict requirements for fabricating films with desired functionality and architecture design.^2,3,9^

Perhaps, the most stringent requirement is the temperature that the flexible substrate has to withstand in order to obtain flexible thin films with the required morphology and architecture designs without degrading their physical properties.^1–4^ For instance, the widely used polymer substrates for growing flexible thin films mostly would result in amorphous or polycrystalline thin films because they usually cannot withstand the high-temperature deposition process. Alternatively, flexible metal substrates might appear to be a viable choice for obtaining thin films with good crystallinity due to their better endurance to higher temperatures. However, it turns out that most metal substrates with high melting point are high density, mostly polycrystalline with a rough surface, containing metallic impurities, and very reactive with intermediary gas during the thin film growth.^2,9^ Very recently, catalytically mediated epitaxy has been successfully demonstrated for growing highly crystalline Si and Ge nanocrystals on van der Waals substrates, such as graphene and hexagonal boron nitride (hBN), by using solid catalysts composed of Ag + Au.^10^ Although this approach may be able to grow 3D metals and semiconductor nanostructures on 2D van der Waals substrates and expand the horizons for electronic and photonic applications, it is yet to be verified whether or not it would work for growing more complex oxides with specific functionalities. In this respect, some flexible ceramics substrates have been considered as viable candidates for growing high-quality crystalline thin films owing to having flat surfaces up to atomic scale.^2,7^ Especially for thin-film heterostructures, the choice of heterostructure design such as bilayer (2–2 system), particulates (0–3 system), and vertically aligned nanocomposite (VAN) (1–3 system) had been achieved with highly crystalline constituent phases. For instance, recently, flexible mica substrate has attracted tremendous attention since it can be easily exfoliated along the (001)-plane to obtain dangling bond-free atomic level flat surface suitable for growing VAN complex oxide with epitaxial quality.^7,8,11–13^ A further unique advantage of mica substrate is that it can withstand much higher temperatures than most polymer or metal substrates, thus is having better opportunity to obtain highly crystalline films.^14–16^

The present work reports the detailed mechanisms leading to the self-assembled heteroepitaxial growth of the three-dimensional (3D) BiFeO_3_–CoFe_2_O_4_ (BFO–CFO) VAN on flexible mica substrate. Previously, the BFO–CFO VAN grown on various rigid ceramic substrates has been illustrated as multiferroic nanocomposite exhibiting strong magnetoelectric coupling effects,^17–20^ which can bring about profound application potential for next-generation electronic devices in fields such as memory, magnetic sensor, and energy harvesting. It has been recognized that the matrix-pillars configuration of the BFO–CFO VAN was enabled mainly *via* the surface energy difference between the interface of substrate/perovskite-structured BFO and substrate/spinel-structured CFO.^15,16^ Consequently, the fabrication of the BFO–CFO VAN system has been stringently limited by the strict requirements of compatible lattice matching and cubic-to-cubic orientation relationships among the two constituent phases and the substrates.^14,18,20–22^ When the lattice mismatch between either constituent phase and the substrate is too large, substantial amount of dislocations and defects are often observed within the resultant films.^21^ Since the mica is 2D material, how the self-assembled BFO–CFO VAN thin-film grown on mica can still lead to highly crystalline epitaxial growth characteristic is interesting by itself. In this study, we found that the introduction of a buffer layer is essential to obtain high-quality flexible VAN thin films. It is anticipated that this route not only may give rise to the unprecedented opportunity for exploring the possible interesting emergent physical properties of the self-assembled perovskite-spinel VAN systems in flexible fashion,^5–8,11,12^ but also offers enormous potential in integrating heteroepitaxy architectures of other flexible functional complex oxides for the next-generation electronic devices.^14,17,23–26^

## Experimental

In the present experiment, the samples were fabricated by the pulsed laser deposition (PLD) method. We used a single mix-phased BFO–CFO target composed of 65 mol% of BFO and 35 mol% of CFO. A KrF excimer laser was employed to ablate the material from the target with an energy of 250 mJ and a repetition rate of 10 Hz. During deposition, the mica substrate was kept at 650 °C and the chamber was filled with oxygen at a pressure of ∼100 mT. Previously, it was found that the perovskite-structured BFO and spinel-structured CFO would naturally have different surface energies when they are simultaneously deposited onto the single-crystalline perovskite substrates, such as SrTiO_3_ and LaAlO_3_. Hence, one may wet the substrate completely, resulting in a layer-by-layer growth mode to form as the matrix, while the other only wets the substrate partially and an island growth mode prevails to form as pillars.^14–16,18,20,21,25^ In most cases, one expects that the self-assembled growth can only occur when the rigid perovskite substrate being used owing to the necessity of achieving a cubic-to-cubic relation between constituent phases and substrates.^14–16,24^

**Fig. 1:**
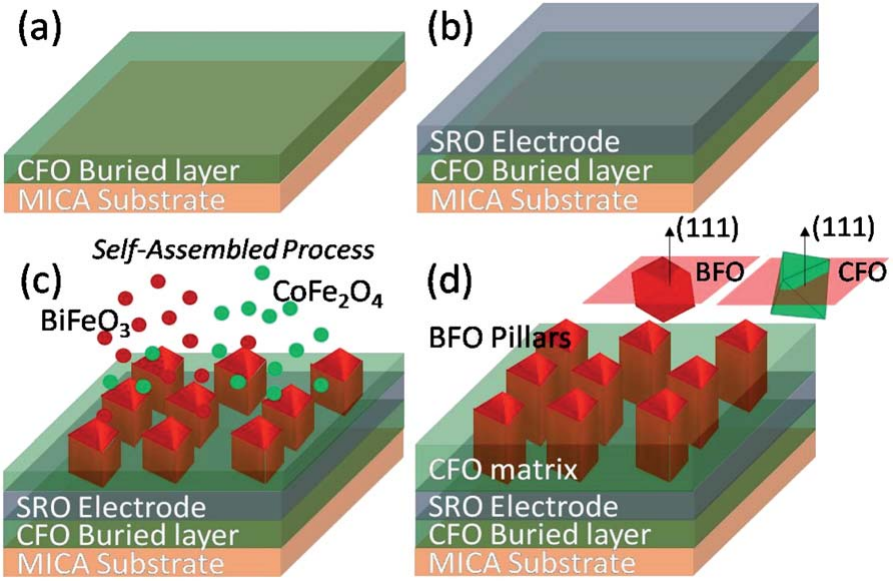
The fabrication process of self-assembled BFO–CFO VAN on a flexible mica substrate. The film deposition is started from (a) CFO buried layer on mica substrate, follwed by (b) SRO bottom electrode, and lastly, (c) the self-assembled BFO–CFO VAN. (d) is the final form of BFO–CFO VAN consist of CFO matrix and BFO pillars.

In contrast, since the mica substrate is having a monoclinic crystal structure with *a* = 0.5199 nm, *b* = 0.9027 nm, *c* = 2.0106 nm, and space group of *C*2/*c*, apparently it is impossible to meet the requirement of cubic-to-cubic relation between constituent phases and substrates. Yet, as mentioned above there have been many successful examples in growing various functional oxides on muscovite mica substrate epitaxially.^7,8^ In the present study, we found that, in order to integrate the electrodes for electric property measurements, it is necessary to introduce an interfacial CFO buffer layer and a SrRuO_3_ (SRO) electrode layer on the 2D mica substrate as the template for growing the self-assembled 3D BFO–CFO VAN. Thus, a layer of CFO was deposited on mica prior to growing an SRO electrode and subsequent BFO–CFO VAN deposition, as depicted schematically in Fig. 1a–d. This recipe is similar to that used for obtaining BFO–CFO VAN on a silicon substrate, wherein, the interfacial buffer layer plays an important role to achieve the self-assembled VAN.^27^ The crystalline phase and structural morphology of the samples were characterized by using atomic force microscopy (AFM, Veeco Multimode 8, under ScanAsyst mode), X-ray diffraction (XRD, Bruker D2 X-ray diffractometer equipped Cu Kα_1_ radiation (*λ* = 1.5406 Å), and the transmission electron microscopy (TEM, FEI Tecnai F20 equipped with high-angle annular dark-field detector). X-ray photoelectron spectroscopy (XPS, ULVAC PHI5600) was employed to reveal the electronics structure of the samples. The magnetic, electric, and optical properties of the obtained films were examined by using the Quantum Design® magnetic property measurement (Superconducting Quantum-Interference device magnetometer (SQUID)), piezoresponse force microscopy (PFM), and UV-vis diffuse reflectance spectroscopy (UV-vis DRS), respectively.

## Result and discussions

**Fig. 2:**
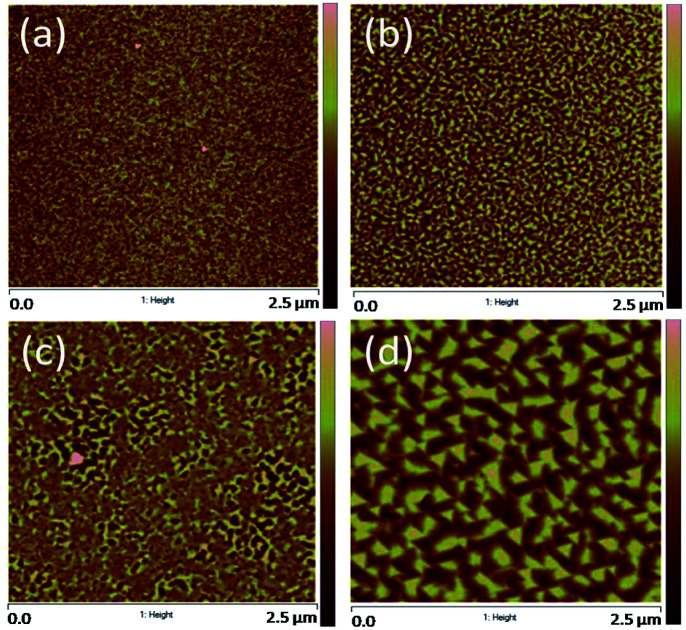
Surfaces morphology of (a) mica substrate; (b) CFO buffer layer on mica; (c) SRO bottom electrode layer on CFO-buffered mica; and (d) BFO–CFO VAN layer on SRO/CFO/mica.

According to our previous report,^28^ CFO is easier to directly grow CFO on mica substrate than BFO does. Thus, we use CFO to serve as an interfacial buffer layer for achieving the so-called quasi van der Waals heteroepitaxy. In fact, it has been demonstrated that this buffer layer played a key role in realizing the self-assembled heteroepitaxy of 3D-like materials on 2D-like substrates.^7,8,29^ However, a more detailed understanding of how the film growth prevails is largely lacking. Here, in order to illustrate how the film morphology evolves as each layer was sequentially deposited, we have taken the AFM images to reveal the surface morphology of the film at each stage of deposition. As depicted in Fig. 2(a) and (b), the surface morphology reveals that the mica substrate and the CFO buffer layer are having a surface roughness of less than ∼1 nm, indicating a well-behaved epitaxial growth of CFO layer on a mica substrate. Considering that the lattice and thermal (at 650 °C) mismatches of mica/CFO is extremely large about ∼35% and ∼70%, respectively, the nearly atomic scale smoothness of the first CFO layer is truly remarkable. In fact, as pointed out by Koma,^30^ high quality heterostructure with very sharp interface and small amount of defects can be achieved even between materials having large lattice mismatch because of the absence of dangling bonds on the clean surfaces of various 2D material substrates. Such growth mechanism has since been coined with the term “van der Waals epitaxy”, because the early stage of the film growth is governed by the van der Waals force. We believe that the 2D nature of the mica substrate could have made the freshly prepared surface free of dangling bonds, therefore mitigated the effects of tremendous lattice and thermal mismatches between mica and the first CFO buffer layer.^7^

For subsequent electrical measurements of BFO–CFO VAN, it is often necessary to integrate a conducting layer to serve as the bottom electrode. In the present study, the SRO was chosen to be the bottom electrode layer since it has a relatively small lattice mismatch with BFO. Moreover, SRO has a pseudo-cubic perovskite structure, hence, is potentially viable in acting as a template for obtaining the self-assembled perovskite-spinel heterostructure. In this sense, the surface morphology of the SRO layer should be of essential importance in determining the quality of the self-assembled VAN to be grown on top of it. Namely, if the surface is too rough, it may promote the growth of BFO and CFO nuclei with random orientation, hence hindering the formation of the desired self-assembled matrix-pillars architecture.^27^ As shown in Fig. 2(c), the surface of the SRO layer remains smooth enough for the subsequent growth of the self-assembled BFO–CFO VAN. The obtained BFO–CFO VAN (Fig. 2(d)) evidently reveals that the triangular-shaped (111)-oriented BFO pillars are embedding within the CFO matrix.

**Fig. 3:**
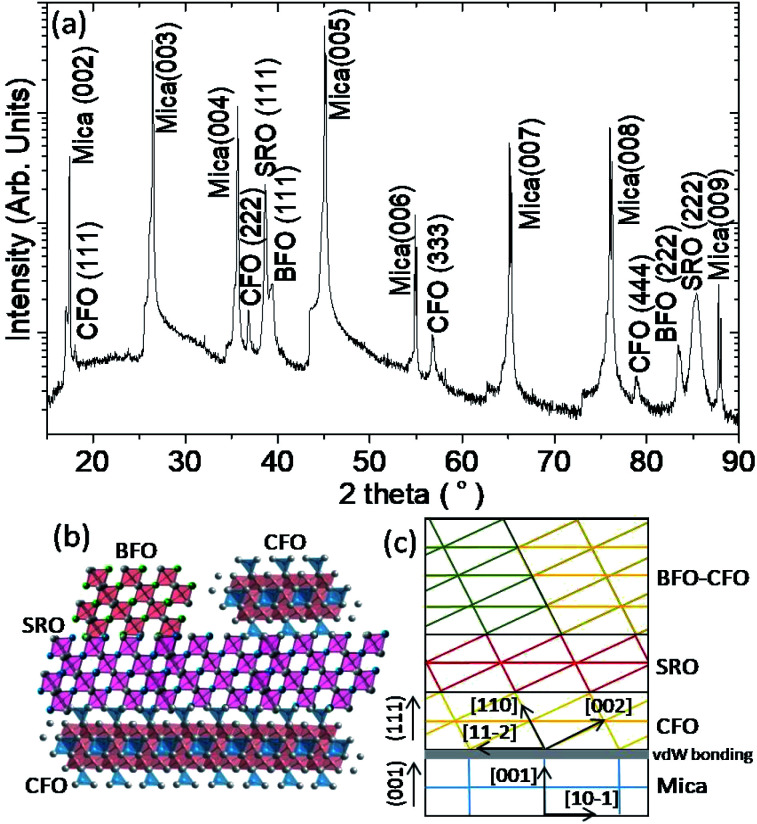
(a) XRD result of BFO–CFO VAN with CFO interfacial buffer layer and SRO bottom electrode grown on a mica substrate. (b) The possible atomic arrangement of the constituent phases on top of mica substrate. (c) The out-of-plane epitaxial relationship indicating the high crystallinity of the constituent phases plays an important role to achieve self-assembled vertically aligned nanocomposite of BFO–CFO phases.

The XRD measurement was conducted to unveil the constituent phases formed in the obtained films and their orientations. According to the XRD results shown in Fig. 3(a), all constituent phases (BFO, CFO, and SRO) were grown along with the (111) orientation on (001)-oriented mica substrate. The inverted cone shape pillars seen in Fig. 2(d)) indeed reflect the (111)-oriented nature of the BFO pillars, while the CFO matrix seems to grow epitaxially on the (111)-oriented SRO layer *via* a layer-by-layer growth manner.^15^Fig. 3(b) and (c) schematically illustrate the possible atomic arrangements of BFO–CFO on top of the CFO and the subsequent SRO layer and how the BFO, SRO, and the BFO–CFO layers stacked during growth processes, respectively. It appears that the first CFO buffer layer not only allows the epitaxial growth of the perovskite structured SRO electrode layer, but also serves as a template similar to the (111)-oriented SrTiO_3_ substrates leading to the formation of the BFO–CFO VAN with BFO pillars and CFO matrix configuration. In this configuration, the CFO apparently has smaller surface energy than BFO does.^15,16^

From the XRD results, we can determine that the *c*-axis lattice parameter of BFO, CFO, and SRO phases are 3.97 Å, 8.52 Å, and 3.89 Å, indicating an out-of-plane strain of 0.12%, 1.79%, and −1.16% with respect to their bulk values, respectively. The strain can depend on factors such as lattice constant and thermal expansion coefficient mismatches.^31^ In the present case, the SRO bottom electrode grown on top of the CFO buffer layer is expected to be under in-plane tensile stress because SRO has smaller lattice constant and larger thermal expansion coefficient than CFO.^32–35^ As for the BFO grown on top of the SRO bottom electrode, a small amount of strain is expected because lattice constant mismatch is small. Therefore, BFO is able to grow epitaxially on top of the SRO layer, even though BFO and SRO are having relatively large difference in thermal expansion coefficient. The XRD result also shows that the CFO phase is under tensile state out-of-plane, presumably due to the fact that the XRD peaks reflect predominantly the contribution from the top-most CFO matrix instead of the underlying CFO buffer layer. In fact, due to the nature of van der Waals epitaxy, the CFO buffer layer might be strain-free from mica substrate, while the top-most CFO matrix is subject to the in-plane compressive strain arising from the SRO bottom electrode because CFO has larger lattice constant but smaller thermal expansion coefficient than SRO.^32–35^

**Fig. 4:**
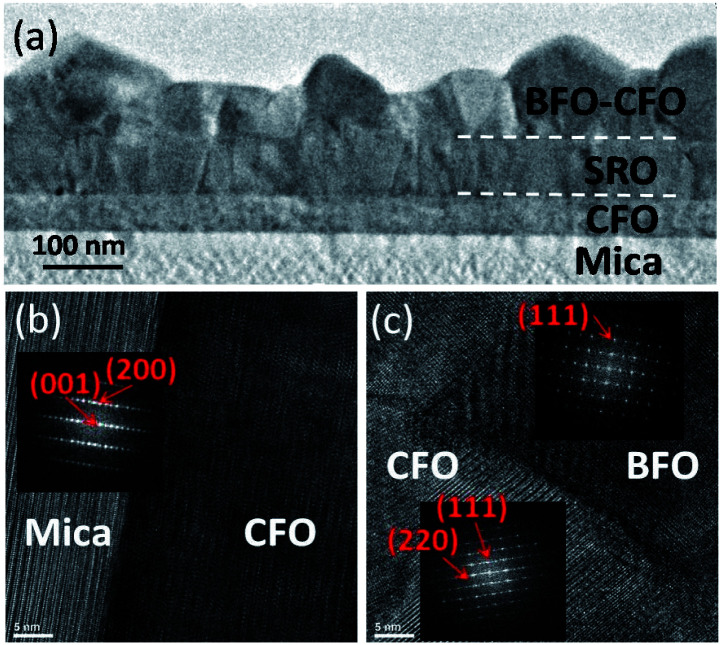
TEM result shows (a) the cross-sectional microstructure of BFO–CFO VAN grown on mica substrate with CFO interfacial buffer layer and SRO bottom electrode. (b) and (c) The high-resolution TEM results with corresponding the fast Fourier transform (FFT) patterns.

Highly crystalline constituent phases hold a key role in achieving high-quality self-assembled VAN heterostructures with desired emergent physical functionalities. In the present case, the atomically flat and dangling bond-free surface of the mica substrate is the origin to achieve the atomic-scale arrangement of the constituent phases, as depicted schematically in Fig. 3(b) and (c). In order to further elucidate that the nanoscale grain structure and morphology is indeed as expected high-resolution cross-sectional TEM analysis was performed. The cross-sectional TEM results are shown in Fig. 4(a) confirms that the BFO pillars are indeed embedded in the CFO matrix, consistent with the conjectures derived from the AFM and XRD results.^15,16^

The TEM results also indicate that, although the SRO layer is apparently polycrystalline, the grain structure seems to be well textured, which is consistent with the XRD results. The highly oriented SRO layer also appears to favor the subsequent growth of the BFO–CFO VAN layer. Moreover, the interfaces among the mica substrate, CFO buffer layer, SRO electrode layer, and BFO pillars-CFO matrix layer all remain very sharp with no discernible sign showing the existence of any secondary phase. The high-resolution TEM results displayed in Fig. 4(b) and (c) again indicate the sharp interface boundaries among various layers. Moreover, the fast Fourier transform (FFT) patterns reveal the epitaxial relationships among the constituent phases are [001]_mica_//[111]_CFO_//[111]_BFO_, in agreement with the XRD results.

**Fig. 5:**
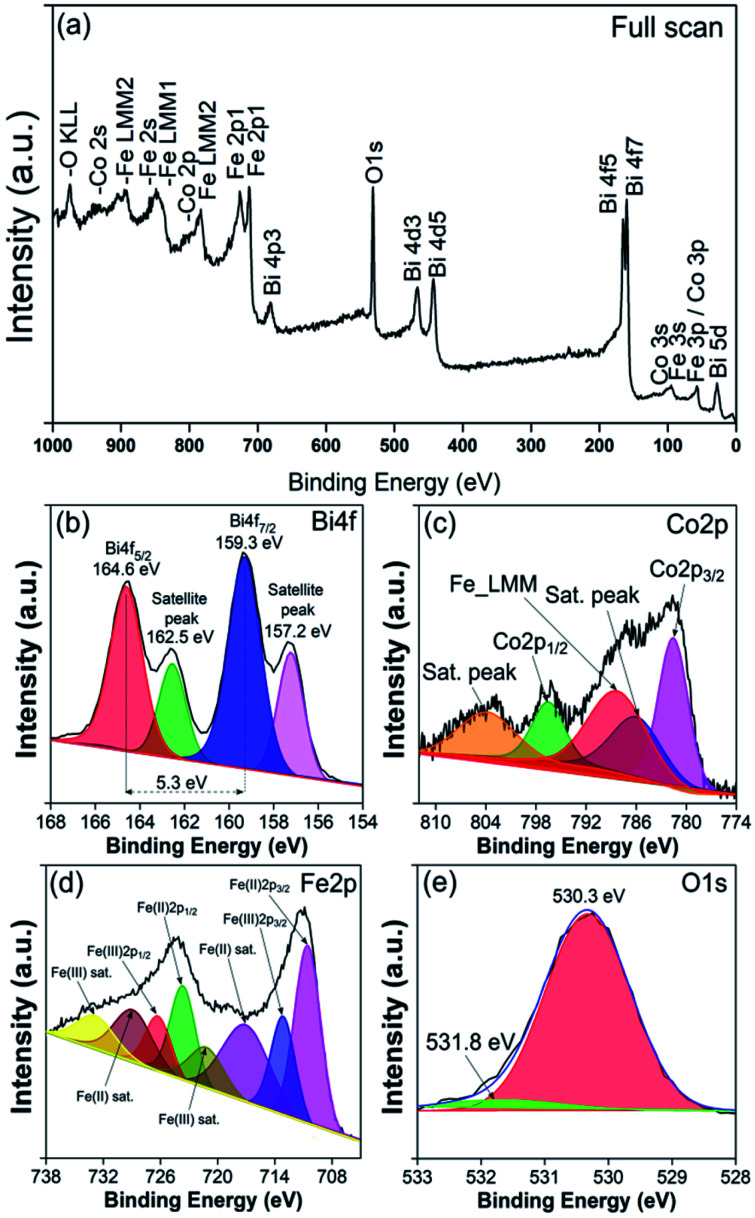
XPS measurements on BFO–CFO/mica sample: (a) full scan; (b) Bi 4f; (c) Co 2p; (d) Fe 2p; and (e) O 1s core level spectra.

To gain more insight into the obtained BFO–CFO VAN on mica substrate, the electronic structures and chemical states of the samples were also measured using XPS. Fig. 5(a) is a full scan of XPS spectra indicating the presence of Bi, Co, Fe, and O elements originating from the constituent phases. Fig. 5(b) shows a closer inspection of the core level XPS spectra, revealing that the Bi 4f core-level spectra are consisting of two major peaks locating at 164.6 eV and 159.3 eV originating from the Bi 4f_5/2_ and 4f_7/2_ electronic states, indicating that Bi is mostly in the Bi^3+^ oxidation state.^36,37^ Nevertheless, it is also evident from Fig. 5(b) that there are also satellite peaks also emerging at 162.5 eV and 157.2 eV, which may be the contribution of spin–orbit coupling peaks of Bi^3+^.^38,39^Fig. 5(c) depicted core level spectra of Co 2p, which consist of main peaks ascribable to Co 2p_1/2_ (796.6 eV), Co 2p_3/2_ (781.6 eV) and satellite peaks (786 eV and 803.9 eV) indicating the existence of Co^2+^ oxidation state.^40,41^

Meanwhile, the Fe 2p core-level spectra shown in Fig. 5(d) can be fitted with three main components: Fe^2+^ 2p_3/2_ (710.6 eV), Fe^3+^ 2p_3/2_ (713.1 eV) and Fe^2+^ satellite peaks.^19,42^ The ratio of Fe^3+^/Fe^2+^ is estimated to be about 2.08. As for O 1s core level spectra (Fig. 5(e)), the peaks located at 530.3 eV and 531.8 eV are associated with oxygen in the metal-oxide lattice and oxygen vacancy (V_O_), respectively.^43,44^ Even though the XRD results do not reveal any signature of secondary phase or even other domain orientations, however, oxygen deficiency presumably might have occurred when the BFO–CFO layer was growing on the SRO layer with apparent texturing grain structure. The ideal atomic ratio of O/Fe from 65% BFO–35% CFO sample is 2.48, while from the experimental data we obtained an O/Fe ratio of ∼2.45, suggesting an oxygen deficiency of about 0.03 (3%) in the BFO–CFO/mica sample. The existence of oxygen deficiency may further induce charge compensation and reduce the oxidation states of Fe ions, namely form formal Fe^3+^ to Fe^2+^.

**Fig. 6:**
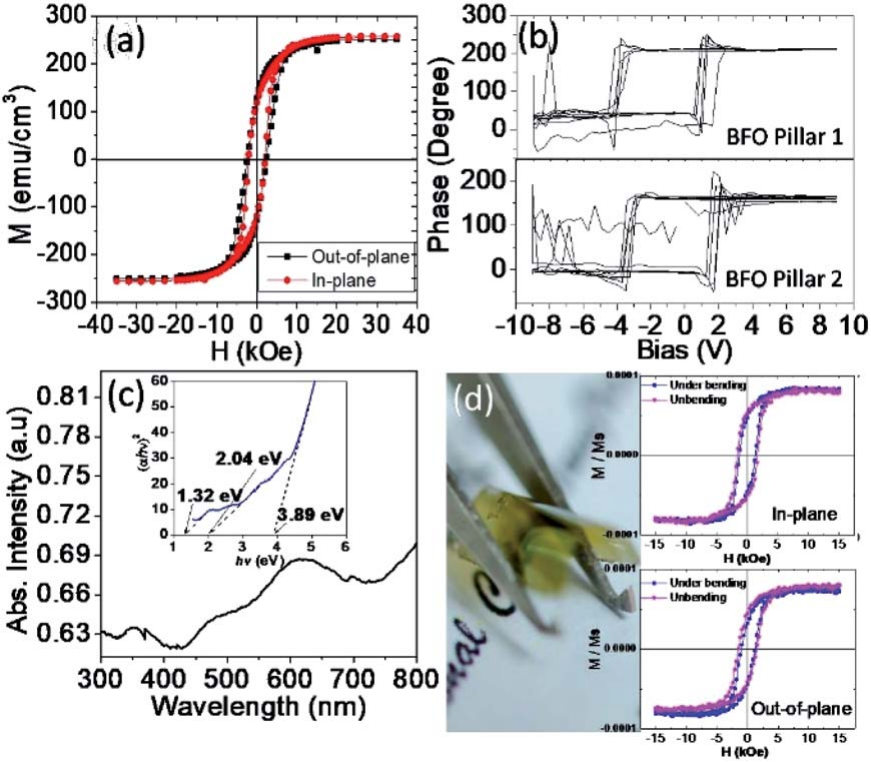
(a) *M*–*H* loops exhibit magnetic isotropy. (b) The *P*–*V* loops were obtained from PFM measurement. (c) The absorption spectra from UV-vis measurement with corresponding Tauc's plot (inset). (d) Photograph showing the flexibility of BFO–CFO/mica, and the magnetic measurements under bending were conducted by applying the magnetic field along in-plane (top panel) and out-of-plane (lower panel) directions.

Next, we shall examine the physical properties of the obtained BFO–CFO VAN, which is strongly affected by the film's structure quality. Fig. 6(a) displays the in-plane and out-of-plane field-dependent magnetization (*M*–*H*) hysteresis of the BFO–CFO VAN samples. It is important to note that the obtained magnetic properties should have been contributed by all constituent phases. For instance, it was evidenced previously that the antiferromagnetic properties of the BFO phase does contribute to the magnetic exchange interaction at the interfaces of BFO and CFO in the BFO–CFO VAN system.^45^ Thus, various energy terms, such as shape and magnetocrystalline anisotropy, as well as the magnetoelastic effect of the ferrimagnetic CFO could all contribute to the global magnetic behaviors observed in the present BFO–CFO/mica sample. Nevertheless, we note that the present results are quite similar to that exhibited by the CFO-phase dominated ferrimagnetic behaviors obtained in the BFO–CFO VAN grown on rigid substrates.^17,45^ Consequently, we speculate that the *M*–*H* loops which exhibit no clear magnetic anisotropy behavior are primarily influenced by the fact that the ferrimagnetic CFO is oriented along the [111]-direction, thus resulting in no shape anisotropy along in-plane and out-of-plane.^18,20^ We believed that, in the present case, the shape and magnetocrystalline anisotropies are more dominant factors as compared to the magnetoelastic effect of CFO because of the relatively smaller strain effect in BFO–CFO/mica compare to the planar CFO epitaxial film grown on rigid single crystalline ceramic substrates.^46^ Furthermore, the electric properties of the samples were tested by the *P*–*V* loops derived from the PFM measurements. As shown in Fig. 6(b), the observed *P*–*V* loops represent the out-of-plane component of the electric polarization originating from the embedded BFO pillars in different regions. Obviously, the BFO pillars preserve most of their ferroelectric characteristics,^23,24^ indicating that the pillars are of excellent crystalline quality. High crystallinity of the BFO and CFO phases is also expected to have better quality interfaces, which, in turn, may further facilitate a strong magnetoelectric coupling *via* strain.

The optical characteristics of the samples were also measured using UV-vis DRS. As shown in Fig. 6(c), the spectra exhibit multiple absorptions in UV (<400 nm), vis (>410 nm), and even in the IR region, suggesting the broadband absorption capability of the present samples. The extrapolation of the Tauc's plot shown in the inset of Fig. 6(c) indicates multiple bandgap values (*E*_g_) of 1.32, 2.04, and 3.89 eV, which can be attributed to bandgap energy of CFO, BFO, and mica, respectively.^47–49^ The results imply that the integration of BFO–CFO on flexible mica substrate seemingly did not significantly affect the optical properties of individual BFO, CFO, and mica phases. Finally, as displayed in Fig. 6(d), the BFO–CFO VAN films grown on mica can be severely bent and appears to be extremely flexible. It is found that the magnetic properties of BFO–CFO/mica is quite robust against mechanical bending. Previous investigations have indicated two types of the magnetic modulation on thin-films grown on flexible mica substrates. Namely, films with robust magnetic properties against mechanical bending and films that are magnetically sensitive to mechanical bending.^28,50–53^ The present results agree with previous work of planar CFO/mica, where the magnetic domain of CFO was retained and relatively immuned from deformation during mechanical bending.^28^ In the planar CFO/mica, the misorientation of CFO is rigid in [111] direction. Thus, the domain wall movement between the domains plays a prominent role in hindering the total magnetization of the sample.^54^ This result also supports our conjecture that the magnetic properties of BFO–CFO/mica were primarily dominated by the CFO phase. It has been also demonstrated that even after more than 1000 bending cycles, the physical properties of the obtained BFO–CFO VAN remained essentially intact.

Such a demonstration clearly indicate that the present study has offered a viable way of developing flexible oxide nanostructures with emergent functionalities for flexible electronic devices, such as magnetic sensor, memory devices, energy harvesting devices, and full-spectrum flexible photovoltaic cell applications.^23,55–59^

## Conclusions

The present study evidently demonstrated that the epitaxial growth of the self-assembled BFO–CFO VAN could be transplanted onto flexible mica substrate without degrading most of the functionalities *via* van der Waals epitaxy of the first CFO buffer layer. We expect that this study might lead to the development of flexible functional oxide materials, and can be used as a role model for developing such complex heteroepitaxy architectures using flexible substrates.

## Author contributions

T. A. prepared the all samples in this study, perform structural and magnetic-electric measurements. A. H. and S. Y. were responsible for XPS and optical measurements. J. Y. J design the study. All authors discussed the results and wrote the manuscript.

## Conflicts of interest

There are no conflicts to declare.

## Supplementary Material

## References

[cit1] Bao Z., Chen X. (2016). Adv. Mater..

[cit2] Liu W., Wang H. (2020). J Materiomics..

[cit3] Zou M., Ma Y., Yuan X., Hu Y., Liu J., Jin Z. (2018). J. Semicond..

[cit4] Sun B., Long Y.-Z., Chen Z.-J., Liu S.-L., Zhang H.-D., Zhang J.-C., Han W.-P. (2014). J. Mater. Chem. C.

[cit5] Nathan A., Ahnood A., Cole M. T., Lee S., Suzuki Y., Hiralal P., Bonaccorso F., Hasan T., Garcia-Gancedo L., Dyadyusha A., Haque S., Andrew P., Hofmann S., Moultrie J., Chu D., Flewitt A. J., Ferrari A. C., Kelly M. J., Robertson J., Amaratunga G. A. J., Milne W. I. (2012). Proc. IEEE.

[cit6] Huang S., Liu Y., Zhao Y., Ren Z., Guo C. F. (2019). Adv. Funct. Mater..

[cit7] Bitla Y., Chu Y.-H. (2017). FlatChem.

[cit8] Wu P.-C., Chu Y.-H. (2018). J. Mater. Chem. C.

[cit9] Ramanujam J., Singh U. P. (2017). Energy Environ. Sci..

[cit10] Periwal P., Thomsen J. D., Reidy K., Varnavides G., Zakharov D. N., Gignac L., Reuter M. C., Booth T. J., Hofmann S., Ross F. M. (2020). Appl. Phys. Rev..

[cit11] Ma C.-H., Lin J.-C., Liu H.-J., Do T. H., Zhu Y.-M., Ha T. D., Zhan Q., Juang J.-Y., He Q., Arenholz E., Chiu P.-W., Chu Y.-H. (2016). Appl. Phys. Lett..

[cit12] Li C.-I., Lin J.-C., Liu H.-J., Chu M.-W., Chen H.-W., Ma C.-H., Tsai C.-Y., Huang H.-W., Lin H.-J., Liu H.-L., Chiu P.-W., Chu Y.-H. (2016). Chem. Mater..

[cit13] Amrillah T., Bitla Y., Shin K., Yang T., Hsieh Y.-H., Chiou Y.-Y., Liu H.-J., Do T. H., Su D., Chen Y.-C., Jen S.-U., Chen L.-Q., Kim K. H., Juang J.-Y., Chu Y.-H. (2017). ACS Nano.

[cit14] Huang J., MacManus-Driscoll J. L., Wang H. (2017). J. Mater. Res..

[cit15] Zheng H., Straub F., Zhan Q., Yang P.-L., Hsieh W.-K., Zavaliche F., Chu Y.-H., Dahmen U., Ramesh R. (2006). Adv. Mater..

[cit16] Zheng H., Zhan Q., Zavaliche F., Sherburne M., Straub F., Cruz M. P., Chen L.-Q., Dahmen U., Ramesh R. (2006). Nano Lett..

[cit17] Oh Y. S., Crane S., Zheng H., Chu Y. H., Ramesh R., Kim K. H. (2010). Appl. Phys. Lett..

[cit18] Amrillah T., Vandrangi S. K., Bitla Y., Do T. H., Liao S.-C., Tsai C.-Y., Chin Y.-Y., Liu Y.-T., Lin M.-L., He Q., Lin H.-J., Lee H.-Y., Lai C.-H., Arenholz E., Juang J.-Y., Chu Y.-H. (2016). Nanoscale.

[cit19] Amrillah T., Chen Y.-X., Duong M. N., Abdussalam W., Simanjuntak F. M., Chen C.-H., Chu Y.-H., Juang J.-Y. (2020). CrystEngComm.

[cit20] Liao S.-C., Tsai P.-Y., Liang C.-W., Liu H.-J., Yang J.-C., Lin S.-J., Lai C.-H., Chu Y.-H. (2011). ACS Nano.

[cit21] Dix N., Muralidharan R., Rebled J.-M., Estradé S., Peiró F., Varela M., Fontcuberta J., Sánchez F. (2010). ACS Nano.

[cit22] Zhu Y. M., Ke D., Yu R., Hsieh Y. H., Liu H. J., Liu P. P., Chu Y. H., Zhan Q. (2013). Appl. Phys. Lett..

[cit23] Catalan G., Scott J. F. (2009). Adv. Mater..

[cit24] Martin L. W., Chu Y.-H., Ramesh R. (2010). Mater. Sci. Eng., R.

[cit25] Guo S., Xu F., Wang B., Wang N., Yang H., Dhanapal P., Xue F., Wang J., Li R.-W. (2018). Adv. Mater. Interfaces.

[cit26] Wang Y., Hu J., Lin Y., Nan C.-W. (2010). NPG Asia Mater..

[cit27] Kim D. H., Aimon N. M., Sun X. Y., Kornblum L., Walker F. J., Ahn C. H., Ross C. A. (2014). Adv. Funct. Mater..

[cit28] Liu H.-J., Wang C.-K., Su D., Amrillah T., Hsieh Y.-H., Wu K.-H., Chen Y.-C., Juang J.-Y., Eng L. M., Jen S.-U., Chu Y.-H. (2017). ACS Appl. Mater. Interfaces.

[cit29] Alaskar Y., Arafin S., Wickramaratne D., Zurbuchen M. A., He L., McKay J., Lin Q., Goorsky M. S., Lake R. K., Wang K. L. (2014). Adv. Funct. Mater..

[cit30] Koma A. (1999). J. Cryst. Growth.

[cit31] Chen A., Hu J.-M., Lu P., Yang T., Zhang W., Li L., Ahmed T., Enriquez E., Weigand M., Su Q., Wang H., Zhu J.-X., MacManus-Driscoll J. L., Chen L.-Q., Yarotski D., Jia Q. (2016). Sci. Adv..

[cit32] Dimakis E., Iliopoulos E., Tsagaraki K., Adikimenakis A., Georgakilas A. (2006). Appl. Phys. Lett..

[cit33] Neumeier J. J., Cornelius A. L., Andres K. (2001). Phys. Rev. B: Condens. Matter Mater. Phys..

[cit34] Yamanaka S., Maekawa T., Muta H., Matsuda T., Kobayashi S., Kurosaki K. (2004). J. Solid State Chem..

[cit35] Zhou J., He H., Nan C.-W. (2007). Appl. Surf. Sci..

[cit36] Quan Z., Hu H., Xu S., Liu W., Fang G., Li M., Zhao X. (2008). J. Sol-Gel Sci. Technol..

[cit37] Das R., Sharma S., Mandal K. (2016). J. Magn. Magn. Mater..

[cit38] Cao F., Wang J., Li S., Cai J., Tu W., Lv X., Qin G. (2015). J. Alloys Compd..

[cit39] Ma Y., Lv P., Duan F., Sheng J., Lu S., Zhu H., Du M., Chen M. (2020). J. Alloys Compd..

[cit40] Zhou Z., Zhang Y., Wang Z., Wei W., Tang W., Shi J., Xiong R. (2008). Appl. Surf. Sci..

[cit41] Li N., Zheng M., Chang X., Ji G., Lu H., Xue L., Pan L., Cao J. (2011). J. Solid State Chem..

[cit42] Kothari D., Reddy V. R., Gupta A., Phase D. M., Lakshmi N., Deshpande S. K., Awasthi A. M. (2007). J. Phys.: Condens. Matter.

[cit43] Bharti B., Kumar S., Lee H.-N., Kumar R. (2016). Sci. Rep..

[cit44] Wei X. Q., Man B. Y., Liu M., Xue C. S., Zhuang H. Z., Yang C. (2007). Phys. B.

[cit45] Chen Y.-J., Hsieh Y.-H., Liao S.-C., Hu Z., Huang M.-J., Kuo W.-C., Chin Y.-Y., Uen T.-M., Juang J.-Y., Lai C.-H., Lin H.-J., Chen C.-T., Chu Y.-H. (2013). Nanoscale.

[cit46] Axelsson A.-K., Aguesse F., Tileli V., Valant M., Alford N. McN. (2013). J. Alloys Compd..

[cit47] Parhizkar J., Habibi M. H., Mosavian S. Y. (2019). Silicon.

[cit48] Gao F., Chen X. Y., Yin K. B., Dong S., Ren Z. F., Yuan F., Yu T., Zou Z. G., Liu J.-M. (2007). Adv. Mater..

[cit49] Kim S. S., Khai T. V., Kulish V., Kim Y.-H., Na H. G., Katoch A., Osada M., Wu P., Kim H. W. (2015). Chem. Mater..

[cit50] Wu P.-C., Chen P.-F., Do T. H., Hsieh Y.-H., Ma C.-H., Ha T. D., Wu K.-H., Wang Y.-J., Li H.-B., Chen Y.-C., Juang J.-Y., Yu P., Eng L. M., Chang C.-F., Chiu P.-W., Tjeng L. H., Chu Y.-H. (2016). ACS Appl. Mater. Interfaces.

[cit51] Amrillah T., Quynh L. T., Nguyen Van C., Do T. H., Arenholz E., Juang J.-Y., Chu Y.-H. (2021). ACS Appl. Mater. Interfaces.

[cit52] Zheng W. C., Zheng D. X., Wang Y. C., Jin C., Bai H. L. (2018). Appl. Phys. Lett..

[cit53] Liu J., Feng Y., Tang R., Zhao R., Gao J., Shi D., Yang H. (2018). Adv. Electron. Mater..

[cit54] Shen L., Liu M., Ma C., Lu L., Fu H., You C., Lu X., Jia C.-L. (2018). Mater. Horiz..

[cit55] Licht S. (2001). J. Phys. Chem. B.

[cit56] Loh L., Briscoe J., Dunn S. (2014). Nanoscale.

[cit57] Guo Y., Guo Z., Zhong M., Wan P., Zhang W., Zhang L. (2018). Small.

[cit58] Ryu J., Kang J.-E., Zhou Y., Choi S.-Y., Yoon W.-H., Park D.-S., Choi J.-J., Hahn B.-D., Ahn C.-W., Kim J.-W., Kim Y.-D., Priya S., Lee S. Y., Jeong S., Jeong D.-Y. (2015). Energy Environ. Sci..

[cit59] Melzer M., Mönch J. I., Makarov D., Zabila Y., Cañón Bermúdez G. S., Karnaushenko D., Baunack S., Bahr F., Yan C., Kaltenbrunner M., Schmidt O. G. (2015). Adv. Mater..

